# Supplementary material for: Geospatial distribution and multilevel determinants of inadequate minimum dietary diversity and its consequences for children aged 6–23 months in Sub-Saharan Africa
Source: PLoS One. 2025 May 7;20(5):e0321254. doi: 10.1371/journal.pone.0321254 (PMC12057906; doi:10.1371/journal.pone.0321254)
Supplement: S1 Table — (DOCX) [file pone.0321254.s001.docx]

Supporting Information file 1 Table: The country included in the analysis using DHS data from 2010-2020

| S.No | Country | Survey year | Survey type | Weighted Sample | Country | Survey year | Survey type | Weighted Sample |
| --- | --- | --- | --- | --- | --- | --- | --- | --- |
| 1 | Angola | 2015/16 | Measure DHS | 2,067 | Malawi | 2016 | Measure DHS | 1,633 |
| 2 | Burkina-Faso | 2010 | Measure DHS | 2,062 | Mozambique | 2019 | Measure DHS | 3,250 |
| 3 | Benin | 2017/18 | Measure DHS | 3,865 | Nigeria | 2018 | Measure DHS | 3,575 |
| 4 | Burundi | 2016/17 | Measure DHS | 1,901 | Niger | 2012 | Measure DHS | 1,554 |
| 5 | DR. Congo | 2013/14 | Measure DHS | 2,567 | Namibia | 2013 | Measure DHS | 649 |
| 6 | Congo | 2011/12 | Measure DHS | 1,488 | Rwanda | 214/15 | Measure DHS | 1,138 |
| 7 | Ivory Coast | 2011/12 | Measure DHS | 1,110 | Sierra Leone | 20189/20 | Measure DHS | 1,440 |
| 8 | Cameroon | 2018 | Measure DHS | 1,366 | Senegal | 2019/20 | Measure DHS | 1,757 |
| 9 | Ethiopia | 2019 | Mini-EDHS | 1,458 | Chad | 2014/15 | Measure DHS | 2,927 |
| 10 | Gabon | 2012 | Measure DHS | 1,159 | Togo | 2013/14 | Measure DHS | 1,062 |
| 11 | Ghana | 2014 | Measure DHS | 847 | Tanzania | 2016 | Measure DHS | 3,014 |
| 12 | Gambia | 2019 | Measure DHS | 1,167 | Uganda | 2016 | Measure DHS | 1,366 |
| 13 | Guinea | 2018 | Measure DHS | 1,036 | South Africa | 2016 | Measure DHS | 428 |
| 14 | Kenya | 2014 | Measure DHS | 2,793 | Zambia | 2018 | Measure DHS | 2,771 |
| 15 | Comoros | 2012 | Measure DHS | 858 | Zimbabwe | 2015 | Measure DHS | 1,593 |
| 16 | Liberia | 2020 | Measure DHS | 848 | Total(SSA) |  |  | 57, 912 |
| 17 | Lesotho | 2020 | Measure DHS | 464 |  |  |  |  |
| 18 | Mali | 2018 | Measure DHS | 2,699 |  |  |  |  |
