# Supplementary material for: Geospatial distribution and multilevel determinants of inadequate minimum dietary diversity and its consequences for children aged 6–23 months in Sub-Saharan Africa
Source: PLoS One. 2025 May 7;20(5):e0321254. doi: 10.1371/journal.pone.0321254 (PMC12057906; doi:10.1371/journal.pone.0321254)
Supplement: Attachment — Submitted filename: Comments MDD.docx [file pone.0321254.s002.docx]

General comments

Although writing an English scientific manuscript is a challenge for many scholars, especially for non-native speakers, I appreciate the authors' efforts. However, there are many grammatical errors and language across the document. As a result in the scholarly community, it is important to maintain the message being conveyed in the manuscript is unambiguous as possible. Hence, your manuscript must be reviewed by a person proficient in written English.

The research topic, **"Spatial Distribution, Multilevel Factors of Inadequate Minimum Dietary Diversity, and its Consequence among Children aged 6-23 months in Sub-Saharan Africa,"** aligns well with the findings of the manuscript. The study effectively addresses the spatial distribution and multilevel factors, and it also you tried to identify significant health consequences of inadequate dietary diversity. However, the consequences are not as elaborately discussed as the other aspects, which might lead to a perception that the focus was more on factors and distribution. If the consequences were intended to be a primary focus, they could be emphasized more in the discussion and conclusion sections to better align with the stated topic. Otherwise I suggest revision of the topic.

Abstract

Abstract, Line 15-16: The phrase "Because of the evidence of geospatial distribution and multilevel factors of inadequate minimum dietary diversity (MDD) and its consequence among children in Sub-Saharan Africa (SSA) remains limited and inadequate feeding practices can lead to malnutrition in young children" need to be rephrased for clarity.

Introduction

The introduction is comprehensive but somewhat dense. While it covers the necessary background, the flow can be improved to enhance readability. Breaking down long sentences

For instance, the first sentence could be split into two: "Adequate minimum dietary diversity (MDD) is crucial for the healthy growth and development of children aged 6 to 23 months. Nutrient adequacy of a diet is influenced by food groupings."

The introduction does a good job of explaining why MDD is important and the consequences of inadequate dietary diversity. However, it would benefit from more specific context about why this study is necessary. What gaps in the existing literature does this study address?

The connection to the SDGs is a strong point but needs to be more explicitly tied to the study's objectives. While the introduction mentions SDG 2, it could be more explicit about how the findings of this study will contribute to achieving these goals.

The introduction uses various statistics to highlight the prevalence of malnutrition and inadequate MDD. Ensure that all statistics are up-to-date and properly referenced.

Ensure all terms and abbreviations are clearly defined upon first use. For instance, while MDD is defined, terms like ANC (antenatal care) and PNC (postnatal care) are not immediately explained. A brief definition of these terms when first mentioned would aid in clarity.

The introduction should aim to engage the reader by highlighting the potential impact of the study. Emphasizing how this research can lead to actionable strategies and policy recommendations will underline its importance.

Methods

Methods, Line 21: Specify the tools or software used for spatial analysis

The manuscript outlines that data were sourced from multiple cross-sectional surveys conducted in SSA in different years. While the nature of surveys are mentioned, there is a lack of detail on how the data from these surveys were harmonized to ensure consistency and comparability. Range of years, information on the survey instruments, sampling methods, and the measures taken to address potential biases in the data collection process would be beneficial. Additionally, details on the specific demographic and geographic variables collected should be provided to understand the data's scope fully.

The study employs a spatial scan statistic to analyze the geographical distribution of your outcome variable in SSA. The chosen methodology allows for the identification of spatial clusters, which is appropriate for the study's aim of detecting geographical patterns. However, additional clarity on the rationale behind selecting this specific statistical method over others would enhance the robustness of the justification. For instance, a comparison with other clustering methods like Bayesian approaches could provide a more comprehensive background for this study.

Discussion

The discussion rightly connects the high prevalence of inadequate MDD to economic factors and healthcare service disparities. However, it would be beneficial to provide more detailed comparisons with specific regions outside SSA to highlight unique challenges or similarities. This can help in understanding whether similar interventions might be applicable or if entirely new strategies are needed.

The spatial analysis is a strong point of the discussion, as it highlights the importance of localized interventions. It would be useful to include more specific examples or case studies from the mentioned countries (e.g., Burkina Faso, Ivory Coast) to illustrate how these problem manifest differently and what specific strategies have been or could be employed effectively.

Line 300-306, you stated that, the low consumption of eggs, fruits, and vegetables is attributed to financial constraints and economic challenges in rural areas, where people might prefer selling nutritious food items like eggs to mitigate economic difficulties. This section should be effectively points out the economic rationale behind dietary choices. To strengthen this point, the discussion could benefit from integrating qualitative data or anecdotes from similar studies that capture the lived experiences of families making these choices. This would provide a more holistic understanding of the barriers to adequate nutrition.

The policy recommendations are crucial, and the call for improved public education is particularly relevant. However, the discussion could be enriched by suggesting specific types of educational programs or policy interventions that have proven successful in similar contexts.
