# Supplementary material for: Geospatial distribution and multilevel determinants of inadequate minimum dietary diversity and its consequences for children aged 6–23 months in Sub-Saharan Africa
Source: PLoS One. 2025 May 7;20(5):e0321254. doi: 10.1371/journal.pone.0321254 (PMC12057906; doi:10.1371/journal.pone.0321254)
Supplement: Attachment — Submitted filename: Authos Response.docx [file pone.0321254.s004.docx]

**Authors’ Responses to Editor and Reviewers Comments**

**Dear Academic Editor of PLOS One**

“**Geospatial Distribution, Multilevel Factors of Inadequate Minimum Dietary Diversity, and its Consequence among Children aged 6-23 months in Sub-Saharan Africa**”

Dear PLOS One Editors and Reviewers;

We are thankful for your constructive comments. We have looked at the comments and have revised our paper accordingly. We hope our paper improved as a result of incorporating the reviewer’s and academic editor’s comments and suggestions. Here are the authors’ responses to the comments.

Please find for your kind consideration the following:

1. A revised manuscript without track changes.
2. A revised paper with tracked changes
3. A rebuttal letter that responds to each point raised by the academic editor and reviewer.

The point-by-point responses of authors are written by hoping these changes would meet with your favorable consideration, we are happy to hear if there are more comments and suggestions. Please do not hesitate to let us know if you have any questions.

Yours Sincerely

Mr. Abel Endawkie Correspondence Author

Department of Epidemiology and Biostatistics School of Public Health College of Medicine and Health Science Wollo University Dessie Ethiopia

Tel. 251935459310	Email address abelendawkie@gmail.com

We have tried our best to improve it accordingly:

Please revise the manuscript.

Journal requirements:

**Point-by-point response of Authors for editor's and reviewers' comment**
**Editor's comments to the Authors**

Journal requirements:

When submitting your revision, we need you to address these additional requirements.

1. Please ensure that your manuscript meets PLOS ONE's style requirements, including those for file naming. The PLOS ONE style templates can be found at

https://journals.plos.org/plosone/s/file?id=wjVg/PLOSOne_formatting_sample_main_body.pdf andhttps://journals.plos.org/plosone/s/file?id=ba62/PLOSOne_formatting_sample_title_authors_affiliations.pdf

**Response:** We thank you and appreciate your prompt and timely response. To ensure compliance with PLOS ONE's formatting guidelines, we have meticulously followed the typesetting requirements for references, tables, and technical specifications for figures and to expedite the process and prevent any delays.

**Reviewer comment for Authors**

**General comments**

Although writing an English scientific manuscript is a challenge for many scholars, especially for non-native speakers, I appreciate the authors' efforts. However, there are many grammatical errors and language across the document. As a result in the scholarly community, it is important to maintain the message being conveyed in the manuscript is unambiguous as possible. Hence, your manuscript must be reviewed by a person proficient in written English.

**Response:** Dear reviewer; we are great full to thank for your kind, prompt, and timely response. To the maximum effort of authors and grammar and spelling checkup, we tried to correct your suggestion of English writing of scientific manuscript.

**Comment 1:** The research topic: **"Spatial Distribution, Multilevel Factors of Inadequate Minimum Dietary Diversity, and its Consequence among Children aged 6-23 months in Sub-Saharan Africa,"** aligns well with the findings of the manuscript. The study effectively addresses the spatial distribution and multilevel factors, and it also you tried to identify significant health consequences of inadequate dietary diversity. However, the consequences are not as elaborately discussed as the other aspects, which might lead to a perception that the focus was more on factors and distribution. If the consequences were intended to be a primary focus, they could be emphasized more in the discussion and conclusion sections to better align with the stated topic. Otherwise I suggest revision of the topic.

**Response 1:** We sincerely appreciate your insightful comment and suggestion and we apologize for any confusion in our writing. Our study focuses on the **Spatial Distribution, Multilevel Factors of Inadequate Minimum Dietary Diversity, and for policy implication we want to highlight the consequence of inadequate minimum dietary diversity among children 6-23 months age. We indicated that in discussion part: Children who did not consume adequate MDD, anemia, stunting, and wasting are significantly associated. This is finding is supported by other study findings (73-75). This may be because children, who do not consume adequate MDD, it suggests that they are not receiving a sufficient variety of nutrients essential for growth and development. Inadequate MDD can contribute to iron deficiency anemia, wasting and stunting in children due to insufficient intake of essential nutrients needed for proper growth and maintenance of body tissues (76).**

**Conclusion: This study indicates that in children who did not consume adequate MDD, anemia, stunting, and wasting are significantly associated. Therefore, improving adequate MDD working at determinants of MDD among children helps to achieve the SDG target 2.2 to end all forms of malnutrition by 2030.**

**Abstract**

**Comment 2:** Abstract, Line 15-16: The phrase "Because of the evidence of geospatial distribution and multilevel factors of inadequate minimum dietary diversity (MDD) and its consequence among children in Sub-Saharan Africa (SSA) remains limited and inadequate feeding practices can lead to malnutrition in young children" need to be rephrased for clarity.

**Response 2:** We would like to thank you for your deep comment. We appreciated your efforts in reviewing the content. We would like to apologize for we confused you in writing. We had revised and corrected it.

**Introduction**

**Comment 3:** The introduction is comprehensive but somewhat dense. While it covers the necessary background, the flow can be improved to enhance readability. Breaking down long sentences

**Response 3:** We would like to thank you for your deep comment. We appreciated your efforts in reviewing the content. We would like to apologize for we confused you in writing. We had revised and corrected it.

**Comment 4:** For instance, the first sentence could be split into two: "Adequate minimum dietary diversity (MDD) is crucial for the healthy growth and development of children aged 6 to 23 months. Nutrient adequacy of a diet is influenced by food groupings."

**Response 4:** We would like to thank you for your deep comment. We appreciated your efforts in reviewing the content. We would like to apologize for we confused you in writing. We had revised and corrected it.

**Comment 5:** The introduction does a good job of explaining why MDD is important and the consequences of inadequate dietary diversity. However, it would benefit from more specific context about why this study is necessary. What gaps in the existing literature does this study address?

**Response 5:** We would like to thank you for your deep comment. We appreciated your efforts in reviewing the content. We would like to apologize for we confused you in writing. We had revised and corrected it. Here are the existing evidence and the reason for conducted this research.

Existed evidence: SDG 2 focuses on achieving food security and improving nutrition through universal access to safe and nutritious food to achieve SDG target 2.2 to end all forms of malnutrition (14). However, in SSA, the situation remains contrary to these goals, with statistics showing a rise in malnutrition (15).

Despite the recommendation and significant advances in child nutrition, the prevalence of inadequate MDD is significantly high in SSA and ranged from 67% to 94% in SSA.

Different literatures showed that, the women's education (19-21), the husband's educational status (19-22), child age (20, 23), mass media exposure (19-21), maternal age (23, 24), occupational status (25, 26), wealth index (19-21), antenatal care (22, 27), and residence (26) in were found to be associated with MDD among children aged 6 to 23 months in different countries of SSA.

Importance of study: The evidence of geospatial distribution both within and between countries, along with the multilevel factors of inadequate MDD and its consequence among children aged 6 - 23 months are important to meet the expected SDG 2.2 by 2030 (end all types of malnutrition). It provides space specific evidence, which enables national and sub-national comparisons, tracks changes over time, and identify the high-risk populations. It informs targeted interventions, guides policy decisions, and monitors progress toward nutrition objectives set by the WHO and SDG in Africa.

Gap identified: However, as far as the author searching effort, the evidence of geospatial distribution between/within countries, and multilevel factors of inadequate MDD and its consequence among children aged 6-23 months in SSA are remains limited. Therefore, this study aimed to identify geospatial distribution and multilevel factors of inadequate MDD and its consequence among children aged 6 to 23 months in SSA using recent DHS data.

**Comment 6:** The connection to the SDGs is a strong point but needs to be more explicitly tied to the study's objectives. While the introduction mentions SDG 2, it could be more explicit about how the findings of this study will contribute to achieving these goals.

**Response 6:** We would like to thank you for your deep comment. We appreciated your efforts in reviewing the content. We would like to apologize for we confused you in writing. We had revised and corrected it. As you know our objective is to determine the spatial distribution of inadequate and multilevel factors of MDD and its consequence (the relationship between inadequate MDD and malnutrition among children in SSA which directly tied with SDG 2.2 to end all types of Malnutrition by 2030.

**Comment 7:** The introduction uses various statistics to highlight the prevalence of malnutrition and inadequate MDD. Ensure that all statistics are up-to-date and properly referenced.

**Response 7:** We would like to thank you for your deep comment. We appreciated your efforts in reviewing the content. We would like to apologize for we confused you in writing. We had revised and corrected it.

**Comment 8:** Ensure all terms and abbreviations are clearly defined upon first use. For instance, while MDD is defined, terms like ANC (antenatal care) and PNC (postnatal care) are not immediately explained. A brief definition of these terms when first mentioned would aid in clarity.

**Response 8:** We would like to thank you for your deep comment. We appreciated your efforts in reviewing the content. We would like to apologize for we confused you in writing. We had revised and corrected it.

**Comment 9:** The introduction should aim to engage the reader by highlighting the potential impact of the study. Emphasizing how this research can lead to actionable strategies and policy recommendations will undermine its importance.

**Response 9:** We would like to thank you for your deep comment. We appreciated your efforts in reviewing the content. We would like to apologize for we confused you in writing. We had revised and corrected it.

Here is the importance of the study: Analyzing regional, national, and continental level data on minimum dietary diversity can offer valuable insights for creating contextually relevant strategies and policies. The evidence of geospatial distribution both within and between countries, along with the multilevel factors of inadequate MDD and its consequence among children aged 6 - 23 months are important to meet the expected SDG 2.2 by 2030 (end all types of malnutrition). It provides space-specific evidence, which enables national and sub-national comparisons, tracks changes over time, and identify the high-risk populations. It informs targeted interventions, guides policy decisions, and monitors progress toward nutrition objectives set by the WHO and SDG in Africa.

**Methods**

**Comment 10:** Methods, Line 21: Specify the tools or software used for spatial analysis

**Response 10:** We would like to thank you for your deep comment. We appreciated your efforts in reviewing the content. We would like to apologize for we confused you in writing. We had revised and corrected it. To execute the spatial analysis, the weighted proportions of outcome variables (MDD) with cluster number were computed in STATA version 17 and the spatial analysis was computed using QGIS version 3.28.15, SAT Scan version 9.6, and SAGA GIS version 2.3.2 statistical software.

**Comment 11:** The manuscript outlines that data were sourced from multiple cross-sectional surveys conducted in SSA in different years. While the nature of surveys are mentioned, there is a lack of detail on how the data from these surveys were harmonized to ensure consistency and comparability. Range of years, information on the survey instruments, sampling methods, and the measures taken to address potential biases in the data collection process would be beneficial. Additionally, details on the specific demographic and geographic variables collected should be provided to understand the data's scope fully.

**Response 11:** We would like to thank you for your deep comment. We appreciated your efforts in reviewing the content. We would like to apologize for we confused you in writing. We had revised and corrected it. In the current study, we used the kids records (KR) data set and the dependent and independent variables were extracted at https://dhsprogram.com/ by contacting them through personal accounts after justifying the reason for requesting the data (28). The study utilized Demographic and Health Survey (DHS) data from 33 Sub-Saharan African (SSA) countries available from 2010-2020(the detailed information on the specific DHS survey years and countries include are provided in supplementary material).

**Regarding geographic variables**, geographic information system (GIS) coordinates, were consolidated into a single dataset and the weighted proportions of outcome variables (MDD) with cluster number were computed in STATA version 17 and the spatial analysis was computed using QGIS version 3.28.15, SAT Scan version 9.6, and SAGA GIS version 2.3.2 statistical software.

**Comment 12:** The study employs a spatial scan statistic to analyze the geographical distribution of your outcome variable in SSA. The chosen methodology allows for the identification of spatial clusters, which is appropriate for the study's aim of detecting geographical patterns. However, additional clarity on the rationale behind selecting this specific statistical method over others would enhance the robustness of the justification. For instance, a comparison with other clustering methods like Bayesian approaches could provide a more comprehensive background for this study.

**Response 12:** We would like to thank you for your deep comment. We appreciated your efforts in reviewing the content. We would like to apologize for we confused you in writing. Even though, the choice of spatial analysis (spatial stascan) and other methodology like Bayesian approaches depends on the specific problem and available data. In this case, spatial scan statistic provides a more straightforward and efficient solution for predicting values at observed locations by identify clusters, trends, and spatial relationships which is valuable for pattern discovery and decision-making and informing decision support for resource allocation, and environmental management, while Bayesian approaches are valuable for more complex modeling tasks by incorporating prior knowledge, providing uncertainty intervals and can handle non-normal data and complex dependencies.

**Comment 13:** The discussion rightly connects the high prevalence of inadequate MDD to economic factors and healthcare service disparities. However, it would be beneficial to provide more detailed comparisons with specific regions outside SSA to highlight unique challenges or similarities. This can help in understanding whether similar interventions might be applicable or if entirely new strategies are needed.

**Response 13:** We would like to thank you for your deep comment. We appreciated your efforts in reviewing the content. We would like to apologize for we confused you in writing. We had revised and corrected it. We state that the prevalence of inadequate MDD is higher than other study finding previous study in SSA and other regions with it possible justification. “The prevalence of inadequate MDD in SSA was 80.3%. Notably, all SSA countries exhibited higher inadequate MDD coverage, which is a higher prevalence compared to other previous study findings in SSA (19, 44) and study findings in Indonesia (45) and in Brazil (46). This situation might be attributed to insufficient child health services in the area, including complementary feeding practices (47). Another factor to consider is that food costs can significantly influence dietary diversity (48, 49) as evidenced by the relationship between gross domestic product and MDD consumption in different countries in Sub-Saharan Africa”.

**Comment 14:** The spatial analysis is a strong point of the discussion, as it highlights the importance of localized interventions. It would be useful to include more specific examples or case studies from the mentioned countries (e.g., Burkina Faso, Ivory Coast) to illustrate how these problem manifest differently and what specific strategies have been or could be employed effectively.

**Response 15:** We would like to thank you for your deep comment. We appreciated your efforts in reviewing the content. We would like to apologize for we confused you in writing. We highlighted that, this finding supported in the previous study finding in these countries Burkina Faso (50), Ivory Coast (51), Ethiopia (52), and Liberia (53). This will be tied to various dimensions such as agriculture, market availability, equity of health care, education, and the sub-national healthcare disparities (54). Therefore, it suggests that it is important to have the need for geographically targeted interventions to address the underlying drivers of inadequate MDD in these high-burden areas.

**Comment 16:** Line 300-306, you stated that, the low consumption of eggs, fruits, and vegetables is attributed to financial constraints and economic challenges in rural areas, where people might prefer selling nutritious food items like eggs to mitigate economic difficulties. This section should be effectively points out the economic rationale behind dietary choices. To strengthen this point, the discussion could benefit from integrating qualitative data or anecdotes from similar studies that capture the lived experiences of families making these choices. This would provide a more holistic understanding of the barriers to adequate nutrition.

**Response 16:** We would like to thank you for your deep comment. We appreciated your efforts in reviewing the content. We would like to apologize for we confused you in writing.

You may understand us the economic rationale behind dietary choices is multifaceted.

Let’s delve to the point, people’s food choices are influenced by prices and income. When faced with financial constraints, individuals may prioritize cheaper food options over more nutritious ones. For example, in rural areas, where economic challenges are prevalent, people might opt to sell nutritious food items (like eggs) rather than consume them directly to mitigate economic difficulties

**Comment 17:** The policy recommendations are crucial, and the call for improved public education is particularly relevant. However, the discussion could be enriched by suggesting specific types of educational programs or policy interventions that have proven successful in similar contexts.

**Response 17:** We would like to thank you for your deep comment. We appreciated your efforts in reviewing the content. We would like to apologize for we confused you in writing. Here are the specific interventions. Based on the finding we state that strategies aimed at promoting frequent antenatal and postnatal visits and improving maternal nutrition are crucial for reducing inadequate MDD among children. Special interventions may be required to promote the consumption of fruits and vegetables, such as vouchers to help with the purchase of these foods for poor communities. This study indicates that in children who did not consume adequate MDD, anemia, stunting, and wasting are significantly associated. Therefore, improving adequate MDD by working at determinants of MDD among children helps to achieve the SDG target 2.2 to end all forms of malnutrition by 2030.

**We feel all of you may understand our feelings and interests.**

**We are thankful for all!!**
