# Supplementary material for: Geospatial distribution and multilevel determinants of inadequate minimum dietary diversity and its consequences for children aged 6–23 months in Sub-Saharan Africa
Source: PLoS One. 2025 May 7;20(5):e0321254. doi: 10.1371/journal.pone.0321254 (PMC12057906; doi:10.1371/journal.pone.0321254)
Supplement: Attachment — Submitted filename: Author response.docx [file pone.0321254.s006.docx]

**Authors’ Responses to Editor and Reviewers Comments**

**Dear Academic Editor of PLOS One**

“**Geospatial Distribution and Multilevel Determinants of Inadequate Minimum Dietary Diversity and Its Consequences for Children Aged 6-23 Months in Sub-Saharan Africa"**

Dear PLOS One Editors and Reviewers;

We are thankful for your constructive comments. We have looked at the comments and have revised our paper accordingly. We hope our paper improved as a result of incorporating the reviewer’s and academic editor’s comments and suggestions. Here are the authors’ responses to the comments.

Please find for your kind consideration the following:

1. A revised manuscript without track changes.
2. A revised paper with tracked changes
3. A rebuttal letter that responds to each point raised by the academic editor and reviewer.

The point-by-point responses of authors are written by hoping these changes would meet with your favorable consideration, we are happy to hear if there are more comments and suggestions. Please do not hesitate to let us know if you have any questions.

Yours Sincerely

Mr. Abel Endawkie Correspondence Author

Department of Epidemiology and Biostatistics School of Public Health College of Medicine and Health Science Wollo University Dessie Ethiopia

Tel. 251935459310	Email address abelendawkie@gmail.com

We have tried our best to improve it accordingly:

Please revise the manuscript.

Journal requirements:

**Point-by-point response of Authors for editor's and reviewers' comment**
**Editor's comments to the Authors**

Journal requirements:

When submitting your revision, we need you to address these additional requirements.

1. Please ensure that your manuscript meets PLOS ONE's style requirements, including those for file naming.

**Response:** We thank you and appreciate your prompt and timely response. To ensure compliance with PLOS ONE's formatting guidelines, we have meticulously followed the typesetting requirements for references, tables, and technical specifications for figures and to expedite the process and prevent any delays.

**Reviewer comment for Authors**

Reviewer #2: The paper calculates sub-national estimates for Minimum Dietary Diversity (MMD) for children aged 6-23 months across 33 out of 40 countries in Sub-Saharan Africa using data from the Demographic and Health Surveys from 2010-20. In addition to identifying and mapping these descriptive statistics on an important variable, the authors also seek to identify covariates associated with variations in MDD. This study stands out in that it provides a harmonized methodology for comparison on MDD across such a wide variety of countries in the region, and does so at a subnational level. It is also important in the way it draws attention to the associations between MDD and predictors of undernutrition such as stunting and wasting, thus providing a window to policy solutions rather than just highlighting the problems.

Response 1: Thank you for your positive and encouraging feedback on our study! We greatly appreciate your thoughtful insights and are glad to hear that you found our methodology and findings valuable. Your comments on the significance of our work in highlighting the associations between Minimum Dietary Diversity (MDD) and undernutrition are particularly encouraging. We look forward to your further thoughts on our revisions. Thank you once again for your support!

Comment 1: I have only one minor suggestion, one minor correction to comment on and the remainder are points for discussion on further work. I highly recommend the work for publication, especially seeing how the comments from the previous reviewer have been diligently addressed.

Comment 2: The first minor comment would be that in L81-83 of the motivation, they state that existing studies of this nature 'remain limited', it would be useful if the authors could find some of the closest equivalents and highlight where they have not gone as far as this one does.
Response 2: Thank you for your valuable feedback on our manuscript! We appreciate your suggestion regarding the need to identify and discuss existing studies in our motivation section. We have made revisions to include relevant studies and highlight how our work extends beyond them. Your input has been instrumental in enhancing the clarity of our research. Thank you once again for your support!

Comment 3: Secondly, I think there may be a minor error in the phrasing of the interpretation of the result regarding distance to health facility. In line 295, it says 'longer distance to health facilities was associated with lower odds of inadequate MDD' when the table shows the opposite result - that those who stated that distance to a health facility was 'not a big problem' were less likely to have inadequate MDD. I would suggest you correct this. It seems to have been correctly interpreted in the discussion, so this must have been a typo in an earlier version.

Response 3: Dear Reviewer, thank you for your thoughtful feedback and kind words about our work! We sincerely apologize for the misinterpretation, which does not align with the actual findings. We have revised the results section accordingly based on your recommendation. Thank you once again for your encouraging and constructive comments!

Comment 4: Regarding the results, they seem to be extremely interesting. For example, the detection of the association between inadequate MDD and lower media exposure is helpful from a policy perspective when thinking about encouraging behavioural change if food sources to increase dietary diversity are already available. It would be interesting as part of future work to look at the extent to which MDD is more attributable to issues of availability (e.g. crop growth, market connectivity) versus cultural/social norms. If such factors were added into the analysis that is presented, it could help to determine to what extent it is easier or more difficult for populations to improve their dietary diversity with simple information campaigns versus market expansion/changes to agricultural practices i.e. to what extent MDD inadequacy is a supply versus a demand problem. Global satellite imagery is increasingly available and could be used to examine the association between cropping patterns and MDD (<https://earth.esa.int/eogateway/success-story/predicting-crop-yield-using-planet-data>). It would also be very interesting to incorporate global satellite data on friction (ease/difficulty of travel) or distance to road networks (<https://data.malariaatlas.org/>). Including such additional factors would help to priorities what kinds of supply side constraints could be better prioritized.
**Response 4:** Dear Reviewer, thank you for your encouraging feedback and for highlighting the intriguing aspects of our findings! We appreciate your suggestion to explore the relative contributions of availability and cultural/social norms to inadequate MDD in future research. Incorporating factors such as crop growth, market connectivity, and the use of global satellite imagery could indeed provide a deeper understanding of the complexities surrounding dietary diversity. We agree that analyzing these elements could help differentiate between supply-side and demand-side challenges, ultimately guiding more effective policy interventions. Your suggestions for utilizing satellite data on travel friction and road networks are particularly insightful. We acknowledge the suggestion which is important thought, However the DHS data has no data like crop growth, and market connectivity. Therefore, we will certainly consider these approaches in our future work. Thank you once again for your thoughtful comments!

Comment 5: Finally, one other limitation in the results that I would highlight is that though the relationships that are identified are important, they are also only weakly significant in each case. It is mentioned that data is used across a 10 year period. Perhaps in future work, it may be helpful to refine the analysis to a cross section within a more limited time range so as to reduce noise and allow relationships to come through more strongly. In that sense, I think the date ranges for the analysis should be added to Table 3 for each of the countries. That would be my final minor suggestion.

Response 5: Dear Reviewer, thank you for your thoughtful feedback and kind words about our work! We have revised the results section accordingly based on your recommendation. Thank you once again for your encouraging and constructive comments!
