# Supplementary material for: Geospatial distribution and multilevel determinants of inadequate minimum dietary diversity and its consequences for children aged 6–23 months in Sub-Saharan Africa
Source: PLoS One. 2025 May 7;20(5):e0321254. doi: 10.1371/journal.pone.0321254 (PMC12057906; doi:10.1371/journal.pone.0321254)
Supplement: Attachment — Submitted filename: Author_response_auresp_4.docx [file pone.0321254.s007.docx]

**Authors’ Responses to Editor and Reviewers Comments**

**Dear Academic Editor of PLOS One**

“**Geospatial Distribution and Multilevel Determinants of Inadequate Minimum Dietary Diversity and Its Consequences for Children Aged 6-23 Months in Sub-Saharan Africa"**

Dear PLOS One Editors and Reviewers;

We are thankful for your constructive comments. We have looked at the comments and have revised our paper accordingly. We hope our paper improved as a result of incorporating the reviewer’s and academic editor’s comments and suggestions. Here are the authors’ responses to the comments.

Please find for your kind consideration the following:

1. A revised manuscript without track changes.
2. A revised paper with tracked changes
3. A rebuttal letter that responds to each point raised by the academic editor and reviewer.

The point-by-point responses of authors are written by hoping these changes would meet with your favorable consideration, we are happy to hear if there are more comments and suggestions. Please do not hesitate to let us know if you have any questions.

Yours Sincerely

Mr. Abel Endawkie Correspondence Author

Department of Epidemiology and Biostatistics School of Public Health College of Medicine and Health Science Wollo University Dessie Ethiopia

Tel. 251935459310	Email address abelendawkie@gmail.com

We have tried our best to improve it accordingly:

Please revise the manuscript.

Journal requirements:

**Point-by-point response of Authors for editor's and reviewers' comment**

**Reviewer comment for Authors**

Reviewer #2: Thank you for addressing the comments in the response letter. I would have liked to see a bit more added in the discussion about the other opportunities for future work, but otherwise all the more immediate issues seem to be addressed.

Response 1: Thank you for your positive and encouraging feedback on our study! We greatly appreciate your thoughtful insights and are glad to hear that you found our methodology and findings valuable.

Comment 2: One note is that I left in my previous revision when asked for 'any other comments' was about the figures. I didn't leave those observations in the main body of the comments but they seem to have not reached you. I'm putting them again here:

- Fig 1 - add title

- Fig 2 - rather than stating 'legend' as the title, could you put the name of the variable? e.g. % of child population age XX meeting MDD? Or add a title, with a less detailed legend?

- Fig 3 - likewise, add a title, make legend a bit clearer as to what 'high' 'low' and 'not significant' refer to

- Fig 4 - same comment to provide a title and provide a clearer labelling for the legend. does 100 mean that the location has 100% of the child population meeting MDD?

- Fig 5 - it's not clear if the circles refer to countries or areas within countries. Please also add a title to the plot. Presumably the numbers are showing rankings. The resolution is not super clear, you may want to make sure you save it as a PNG with 300 DPI at least.

- Fig 6 - also needs a title. I think those figure edits will be necessary for publication as many are missing clear legends or titles. Otherwise, congratulations on your work

Response: Dear Reviewer, Thank you for your thoughtful feedback and kind words about our work! I appreciate your observations regarding the figures, and I apologize for any confusion in previous communications. We have revised and provided the title in the main document as outlined by the journal guideline. We have made the necessary revisions based on your recommendations. Specifically, we have added titles to all figures. We believe these edits will enhance the clarity of the figures and are necessary for publication. Thank you once again for your encouraging and constructive comments!
